# Community voices: policy proposals to promote inclusion in academia through the lens of women in science

**DOI:** 10.1038/s41467-022-31616-6

**Published:** 2022-07-13

**Authors:** Sarah A. Teichmann, Muzlifah Haniffa, Jasmin Fisher

**Affiliations:** 1 Wellcome Sanger Institute, Wellcome Genome Campus, Hinxton, Cambridge, UK; 2 Cavendish Laboratory, University of Cambridge, JJ Thomson Ave, Cambridge, UK; 3 Biosciences Institute and Newcastle NIHR-BRC Dermatology, Newcastle University, Newcastle upon Tyne, UK; 4 UCL Cancer Institute, University College London, London, UK

**Keywords:** Science in culture, Computational platforms and environments

## Abstract

Diversity is a creative force that broadens views and enhances ideas; it increases productivity as well as the impact of our science, making our respective organisations more agile and timely. Equality of opportunity is a key to success for any research organisation. Here we argue that every research organisation, whether in academia or in industry, needs to have better inclusion policies to harness the benefits of diversity in research. Drawing from our personal experiences and perspectives as women in science, we share our suggestions on how to promote inclusion in academia and create a better research culture for all. Our shared experiences highlight the many hurdles women in science face on a daily basis. We stress that rules and regulations, as well as education for awareness, will play critical role in this much needed shift from a male-dominated scientific culture that dates from Victorian times to a modern focus on gender equality in science. The key ingredients of this new culture will be flexibility, transparency, fairness and thoughtfulness.

As three women with leading roles in academic research labs in the UK, we have had many discussions about some of the challenges faced by women in science. We quickly realised that many of our experiences overlapped, and that we had come to many of the same ideas about the root causes of, and potential solutions to, these problems. In this article, we wish to use past experiences (primarily before our current positions) as a springboard for moving forwards in a positive way, in the form of concrete and simple policy proposals that funders and employers can adopt to promote the inclusion of women in science (these proposals are collated in Table 1). We believe that a more inclusive environment will result in better science, and the whole of society will benefit.

**Table 1:** Policy proposals for more inclusive science.

Workplace environment and gender balance	Provide ongoing training in language and unconscious bias.
	Require full transparency around the process of promotion and relative pay.
	Insist that female voices in meetings and panels are equally heard.
	Attempt gender parity wherever possible in all panels, meetings, public recognitions etc..
	Require female representation in collaborative grant applications.
Conflicts of interest, independence and mentorship	Provide personal conflict-of-interest declarations when applying for grants, reviewing for tenure, providing references.
	Require sponsors to sign commitments supporting trainees’ independence to carry on their research direction once a fellowship has ended.
	Require anonymised, verbatim statements of mentorship quality from trainees for any large grant or promotion.
Pregnancy and childcare	Technician support for PhD students/postdocs for the end of pregnancy and periods of maternal or parental leave.
	Require conferences to be accessible for pregnant women and parents of young children.
	Schedule conferences or other mandatory work events on weekdays during working hours.
	Allow flexible working patterns for parents of young children, single parents.
Menopause	Offer statutory compassionate leave and flexible hours to menopausal women where they need it.
	Educate staff about the menopause as part of diversity/inclusivity training.

Even today, we continue to encounter attitudes in science according to which female underrepresentation at higher levels is due to differences in innate ability rather than discrimination and the obstacles women face. Such attitudes were astutely deconstructed in 2006 by Ben Barres^1^, who, as a transgender man who transitioned midway through his career, had a unique perspective on the perception of the influence of gender in the scientific enterprise: “When it comes to bias, it seems that the desire to believe in a meritocracy is so powerful that until a person has experienced sufficient career-harming bias themselves they simply do not believe it exists”.

In our view, the behaviours we experienced and witnessed are permitted and incubated by social norms and institutional structures. While we applaud many of the inclusion initiatives adopted by various organisations in recent years, we think such initiatives work best when they include incentives for behavioural change: rather than asking people to behave well or vaguely arguing for a change in ‘research culture’, it will be more effective to organise the system so that negative behaviours are disincentivised and positive behaviours are rewarded.

While our article focuses on gender, the intersectional nature of these issues needs to be considered for science to become more inclusive (ethnicity, socioeconomic background, disability, sexuality etc.). The problems with research culture are manifold (for a recent survey from the UK, see Brazil, 2021^2^), and will require joined up thinking to tackle. We hope that our recommendations have the potential for a broader impact beyond gender parity: organisations need to take a broad approach to inclusivity, and our suggestions for gender-based policies are necessarily one part of a greater whole.

Starting with the experiences of women in the workplace, we stress the positive impact of behavioural competency frameworks (BCFs) when adopted by institutions. We then address how gender interacts with independence and mentorship before discussing pregnancy, childbirth and the still infrequently discussed topic of the menopause. Throughout, the reader will see certain themes repeatedly arise: the need for flexibility, transparency, fairness and thoughtfulness to make science more inclusive. One of the big lessons from the COVID-19 pandemic is that organisations can cope with dramatic changes in working practice while continuing to remain productive. The system certainly seems to be flexible enough to take on board many of the suggestions we discuss.

## Behavioural competency frameworks to enshrine positive behaviour

Bias, discrimination and abuse still exist in the scientific workplace and cause substantial harm to women’s lives and careers^3,4^. We have collated some of our own experiences of such bias in the Supplementary File: we hope these resonate with some readers and open the eyes of some others. In our discussions, we have repeatedly circled back to the important role that institutions and organisations can play in counteracting such bias—and we have collated some positive examples of good practice in Table 2.

**Table 2:** Examples of positive actions from organisations.

Organisation	Action
European Research Council	Allows an 18-month extension for each childbirth for the mother, and the time taken for paternity leave for the father Beneficiaries of ERC grants must take all measures to promote equal opportunities between men and women, aiming for a gender balance at all levels of personnel.
Wellcome	When a Wellcome Career Development Fellow goes on leave, salary will be provided to extend the junior staff on the project to keep things running.
EMBO, ASCB and others	These organisations run conferences that provide some level of childcare arrangement (e.g. see https://www.embo-embl-symposia.org/info_participants/childcare/index.html).
Nature conferences, Wellcome conferences and others	These organisations have implemented efforts to ensure better representation in the speaker list of conferences (e.g. see https://www.nature.com/articles/d41586-021-03735-5 and https://coursesandconferences.wellcomeconnectingscience.org/wp-content/uploads/2021/03/Wellcome-Connecting-Science-courses-and-conferences-Gender-Balance-Policy.pdf).
Horizon Europe	Uses gender balance as a ranking factor when comparing otherwise similar proposals (see https://ec.europa.eu/research/participants/docs/h2020-funding-guide/cross-cutting-issues/gender_en.htm), and will soon require all grant applications to include a Gender Equality Plan (https://ec.europa.eu/info/research-and-innovation/strategy/strategy-2020-2024/democracy-and-rights/gender-equality-research-and-innovation_en).
Howard Hughes Medical Institute	Postdoctoral sponsors in the HHMI Hannah Gray Fellowships, which aim to recruit and retain underrepresented in the life sciences, are expected to foster a path to independence for the fellow (see https://www.hhmi.org/programs/hanna-h-gray-fellows-program).
Wellcome Sanger Institute	Employs a Behavioural Competency Framework (https://www.sanger.ac.uk/about/careers/life-at-sanger/) which defines how success is measured and the standards of behaviour necessary to build an inclusive community A number of family-friendly and carer-friendly policies including paid leave for carers, enhanced maternity and shared parental leave, and grants for returning from extended absences (https://www.sanger.ac.uk/about/equality-in-science/working-environment/).
University College London (UCL)	Programme to mitigate the gendered impacts of COVID-19 on the careers of staff, particularly those with caring responsibilities.
UCL Cancer Institute	Ringfenced Extended Leave Support Fund to help support personnel and their projects during times of extended leave including parental leave Carers Fund: funding to help support staff and students with caring responsibilities i.e. payments to help pay for childcare to enable attendance at conferences.

Note this is not an exhaustive list but reflects some of the positive changes we have observed

One positive step forward that all organisations can take is to adopt a BCF. A BCF specifically sets out how staff members are expected to behave when employed by an institution. Where rules and norms are unwritten, women may feel that their views have less weight. Putting the norms down on paper will also help people address their own conscious and unconscious behaviours and biases. Two of us have had good experiences of the utility of the BCF of the Wellcome Sanger Institute in promoting inclusive behaviour, and many of the suggestions in the following sections can be enshrined in an organisation’s BCF.

### Women in the workplace: assumptions, ambitions, and men-dominated spaces

Women commonly face assumptions in the scientific workplace, from the way they should dress if they want to succeed as a scientist, to the way they should behave, and we are no exception (see Supplementary Information). We have encountered assumptions based on seniority, as presumably have most established women in science. In a meeting room full of men, we have often automatically been assumed to work in administration or human resources, or if not, at least in some kind of junior role. Similarly, assumptions have been made on the basis of financial independence—where women are equal or main breadwinners but were not perceived as such. Such implicit assumptions conform to what is thought to be the societal norm.

There is a need for a culture change in attitude towards women via ongoing effective diversity training about language and unconscious biases. Importantly, women at all career stages should have standard channels for complaints, or even better, suggestions for policy changes, to be made without fear of retribution. Annual surveys of how women feel in each workplace will help the leadership understand which cultural changes are necessary.

Women in science repeatedly face problems relating to ambition: women being assertive and showing ambition are often perceived as and criticised for being aggressive in contrast to how men assertiveness is perceived. We suggest that promotion should be an open and transparent process which employees can request proactively and encouraged to apply, and not a ‘gift’ from manager to employee but an acknowledgment of the employee’s progress. On the flipside, if women are less assertive and less confident in pushing for promotions, they risk being left behind. The onus needs to be on organisations to counterbalance these tendencies, and promote women in a pro-active top-down way to prevent the gender pay and status gap from widening. One suggestion is to do an annual check on whether women are progressing at the same rate as their comparable and equivalent men counterparts, both in terms of salary and status (i.e. promotion) and in terms of resource and lab space in core-funded institutes.

In many academic workplaces, over-representation of men has negative consequences for women. A woman in a room full of men will often struggle to make herself heard in discussions. This can be simple to remedy: meeting chairs have a responsibility to ensure everyone in a meeting has the opportunity to present and speak, and ensure what they say gets equal discussion and respect. It should be part of an organisation’s culture that it is unacceptable to speak across another person when they are making their point – this could be written into the BCF, for instance.

Men-dominated meetings can create an atmosphere of a ‘boys’ club’, also present in conferences and other settings. These men-dominated spaces can be uncomfortable to women, who may encounter outright sexism, lack of respect or indifference, or difficulties in finding common ground in topics of social conversation. One obvious practical solution is to insist on attempting gender parity where possible in meetings.

Many of the issues of women in the workplace could be helped by the existence of a women’s network and/or a dedicated women’s officer/ombudsman/affairs advisor in organisations. Organisations should support independent women employee networks and support groups that will in turn feed back into the leadership with suggestions for structural and cultural changes. J.F. has personal experience of the effectiveness of a women’s network in a men-dominated field (computer science), and we would recommend all organisations consider establishing and supporting them.

Gender balance is crucial at all levels of the scientific enterprise. For funders, one key area of focus is on the allocation of grants. We have seen many examples of men-only grant consortia where there are obvious women who have expertise that would be complementary, yet they have not been invited to join by the ‘club’. One solution would be that granting agencies only accept collaborative grant applications with at least one woman in it or with gender balance in personnel (as in the case of the European Research Council, see Table 2), or at least a justification for why this was not possible (e.g. for cases where women were invited to join but declined). On a related note, where scientists are highlighted through recognition in the form of naming of institutes, buildings, streets, award lectures, etc., an even distribution with regards to gender balance should be aimed for to overcome existing imbalance of male-dominated recognition. Scientific role models are incredibly important, and we should be aiming to promote a diverse set that everyone can aspire to.

### Conflicts of interest, independence and mentorship

As in any other workplace, interpersonal relationships are going to occur in scientific institutions. However, when undeclared, such relationships can have negative consequences for the people around them, and predominantly negatively affect women. Relationships that cross seniority—and this most commonly involves a senior man and junior woman—can affect the environment of a whole lab, as shared in an experience in the Supplementary File. We believe that requiring senior staff members and grant awardees to declare personal conflicts-of-interest statements, much as they would for financial conflicts, would solve a lot of these problems. You wouldn’t be allowed to write a reference letter for your child, so why should you be allowed to do one for your partner? The fact that universities often do not articulate this means that the environment is permissive to exploitation of this type of situation. A more widespread use of conflict-of-interest declarations would help bring transparency to the research environment, which in turn would help nurture younger researchers, men and women alike. Behaviour around relationships can also be written in to the BCF.

An important aspect of mentoring and support involves independence. For example, once a postdoctoral researcher finishes a fellowship, it is common practice for the supervisor to take hold of the project, and often even explicitly forbid the younger researcher from continuing their work. This can stunt the researcher’s progress, particularly if they have spent many years on a research question or topic they are deeply invested in. When it comes to personal fellowships that the postdoc brings into the lab, there should be clarity around what research direction the junior scientist will pursue independently after the fellowship duration, and which directions the supervisor will maintain in their lab. But again, rather than just try to persuade, we believe concrete action will provide stronger incentives: sponsors could for instance be required to make a statement or sign a document guaranteeing the fellow’s freedom to continue the work once the fellowship ends.

Another aspect of mentorship that we believe could be better formalised relates to evidence. For trainees, a letter of support from an experienced sponsor is vital for them to gain funding for their future research. But for senior figures, evidence of their competence in mentorship is often not required for reviews or assessments, or if it is, the Principal Investigator fills in that part of the application for themselves. The risk of course is that labs that foster toxic environments are permitted to continue in the same vein: as long as publications and grant money keep on coming, the wellbeing of the people doing the research can be discarded. Without negative feedback, abusive or toxic behaviour in science will continue, behaviours we have all experienced to various degrees. We suggest that statements of mentorship from trainees could be integral to any large grant or application or tenure/promotion. Such statements should of course be anonymous to avoid reprisals from the PI, and thus could be handled by the university’s HR department. This suggestion may provide a way not only to disincentivise bad behaviour but also to recognise and reward good mentors.

Declaring all types of conflict-of-interest, independence and evidencing mentorship would act as incentives for positive behaviour in Principal Investigators, steering them towards acting in a more inclusive and fair manner. Readers need not necessarily wait for the funders to enact these changes: M.H. has started to implement direct evidence of mentorship in her own applications for funding regardless of whether the funders request it.

### Pregnancy and childcare

Pregnancy and childcare lead to unique challenges for women in science^5^. These challenges often come at critical early stages of their career—they might be finishing a PhD, or in the early years prior to having a permanent position where there is acute pressure to publish and bring in grant money. Inadequate support during this period can therefore negatively impact the woman’s career as well as her wellbeing, and science as a whole loses out. In the examples recounted in the Supplementary Information, pregnancy circumstances and childcare duties were not properly taken into consideration by superiors/funders, and there was a distinct lack of empathy.

We have repeatedly encountered the following attitude in science: that pregnancy is a problem rather than something to celebrate and support. In our view, this attitude is partly a result of the lack of structural support built into research organisations. We believe that issues around pregnancy could also be integrated into mandatory management or inclusivity training for Primcipal Investigators. Supervisors themselves must consider which tasks should be assigned to pregnant women, showing sensitivity in terms of practicality, but also in terms of the potential emotional toll of tasks like embryo dissection. Pregnant women need a suitable and safe working environment, with limited hours standing, parking spaces that are not too far from the workplace, adequate breaks, and good food and drink options. At the same time, it is important to emphasise that the physical experience of pregnancy varies widely, and some women will be able and want to work throughout and should be allowed to opt for this route. Empowerment and flexibility are both key in this context.

Women who have just given birth are in a vulnerable position due to recovery from the birth and the demands of looking after the new-born baby. As the example of our colleague in Supplementary Information clearly shows, institutional or funder reviews should include clear policies taking into account maternity leave and the kinds of extenuating circumstances that come along with childcare and could offer choices of dates to accommodate. When external reviewers for grants or promotion are assessing a scientist’s output, at a bare minimum they should take into account time taken on parental leave. All of these things should be explicitly written into the reviewer guidelines.

In a broader sense, funders and organisations must take into account the physical reality of pregnancy, birth and breastfeeding. One simple suggestion, already adopted by many institutions including the Wellcome Sanger Institute, is to automatically extend the tenure track period when parental leave is taken. This would take account of the disruption to work and the resultant drop in productivity (i.e. in terms of publications) that has been recently quantified^6^. On a related note, we believe that contract extensions and leave for miscarriages and stillbirths should be enshrined in organisational policies, given their physical and mental toll on mothers.

When a scientist takes time away from the lab when pregnant or parental leave, this can often put the brakes on their research projects. Returning from leave, it can often take many months to get projects back up off the ground. Thus there is a need for experimental support during this period, for instance to keep cell lines or data collection running. Some funders already provide this (see Table 2). We feel that extending such experimental support to PhD students and postdocs would be game-changing in supporting the development of their research. It will also take away one of the unfair obstacles to relatively younger women scientists having children.

Overall, these issues speak to a general need for a long-term perspective when evaluating researchers that takes into account extensions and interruptions. Importantly, these extensions should be automatically granted for common scenarios such as childbirth, where a standard framework can be used, rather than being decided ad hoc by heads of departments or institutes. In this way, a contract extension becomes a right rather than a favour granted by an individual.

It is reasonable to expect that all modern places of work would be welcoming to new mothers when they decide to return to work, and provide a flexible environment for feeding. Luckily, at least in the UK, the situation has improved and the law requires workplaces to provide “staying in touch days”, provisions for expressing milk or breastfeeding etc. As a minimum, workplaces should have private spaces for breastfeeding mothers to pump milk. In addition, all parents will benefit from flexible working hours to deal with childcare arrangements. For single parents, such flexibility should be in place essentially until the children leave home, given the challenge of parenting alone. Similarly, employers should recognise that workers with caring responsibilities require particular flexibility.

Having children often coincides with a time in a woman’s career where attending conferences is vital to make connections and promote their work, but in-person conferences pose particular problems especially for those with young children or those who are single parents. Conferences should provide, or at least make available, nursery facilities, and travel bursaries could be established to allow parents with young children to travel with their partner or to cover nursery fees. This would compensate the combination of academic salaries being somewhat modest in the early years when people tend to become parents. Conference programmes should be respectful of needs of parents with young children attending (for instance providing family accommodation, meals, breaks in timetable, reasonable time schedules, location for ease of travel). For mothers who can’t or don’t wish to leave their children, virtual attendance can help, especially now the infrastructure is in place from the pandemic.

One simple (yet often overlooked) policy to make conferences—virtual or in person—more family-friendly is to restrict them to weekdays, allowing parents time with their families on the weekends. The respect for time off schedule work events at weekends is standard in many other work environments, while it seems to be built into the biomedical research culture that there are scheduled work events on weekends. This means that weekend (and also evening) work becomes a necessity rather than a choice, which is a constraint that we should consider reversing in the community.

Finally, it is worth noting here how the unequal effects of childcare on women’s careers was manifested in the COVID-19 pandemic. The burden of childcare, including home-schooling, disproportionately affected women^7,8^. The long-term effects of this have yet to be seen but it is crucial that funders and organisations take it into account when considering the research outputs of mothers during these past two years.

### Breaking the menopause taboo

Menopause is a phase in life that can last several years and often be debilitating, and is still not widely discussed as a obstacle to women’s success in science^9^. While some women will sail through without much trouble, others will suffer daily for years, with negative consequences for their professional life. In contrast to pregnancy and childbirth, menopause tends to affect women already on the academic track, and thus can act against the retention of senior women in leadership roles. In our experience, menopause is still something of a taboo subject, even in academic circles, and we believe it should be discussed much more openly. Structural changes need to accompany this, as organisations regularly fail to take it into account when reviewing performance or allocating tasks and responsibilities.

While treatment options for the effects of menopause are limited, there are ways to deal with it and even come out stronger and wiser at the other end. But this takes time, patience and a lot of consideration. Thus, we argue for the necessity of raising awareness about menopause and its impact, as well as practical steps to allow women that time. These steps include compassionate leave, the possibility to work flexible or reduced hours for periods of time, accepting the need to work from home, and measuring success differently during this period. There is a significant opportunity to explore how the work pattern flexibility resulting from the COVID-19 pandemic can be continued to better support women, and those in childbearing and menopausal life stages in particular. Job sharing in senior roles - including at director’s level - could be offered to better accommodate time constraints. and allow people to continue research and achieve a work-life balance while taking on leadership roles.

A simple suggestion is to formally recognise menopause as a stage of women’s lives to be accommodated by employers as much as pregnancy or childcare. This formal recognition may also help to weaken the taboo on the subject along with the shame, frustration and guilt that ‘women of a certain age’ have to go through. Organisations can additionally play an educative role, including the menopause in the diversity/inclusion training that is increasingly common but generally doesn’t include the menopause at present.

### Getting men on board

All the above discussions relate to a final, important question: how best can we encourage men to be allies in this endeavour? Disincentivising men-only projects is one suggestion. Men should also be confronted with their unacceptable behaviours and made to address them no matter how uncomfortable they find it. For this to work, calling out inappropriate behaviour should not be punished; a comprehensive BCF can be vital in this regard.

But we can also act to incentivise: financial rewards for good behaviours such as mentorships, collaborations with women etc. would be a promising and complementary avenue. Crucially here, these issues cannot be addressed by women alone. Men have to be proactive to change the culture of the workplace—in this vein we recommend Marion Pepper’s recent plea to male scientists to help create inclusive environments^10^. As a complementary strategy, it is also worth acknowledging and showcasing men who support women, both in terms of the everyday scientific process and where they push boundaries to implement structural changes. For example, Nancy Hopkins has recognised the fantastic efforts of former MIT President Chuck Vest and Provost Bob Brown in addressing gender bias at that institution (see https://news.mit.edu/2020/3-questions-nancy-hopkins-improving-gender-equality-in-academia-0930), while here in the UK, we highlight the former MRC Executive Chair Jim Smith, who implemented changes such as the recognition of rights to maternity leave when women switched from one organisation to another. This kind of allyship at the top level makes a massive difference, while on a day-to-day level women very much benefit from smaller acts of support from their men colleagues.

### Conclusion: policies for more inclusive science

In this article, we have used a selection of our personal experiences to illustrate just some of the challenges still faced by women in science. Our main argument is that in order to get rid of many of these obstacles, the right behaviour has to be specifically incentivised, and the structures of science must be reconfigured to ensure fairness. To properly address many of the issues with research culture, appeals to personal behaviour change only go so far. We hope our article encourages anyone reading who holds a leadership role at a funding agency or a research institute that the policy proposals that we argue for (summarized in Table 1) are often relatively simple to implement. A more representative and inclusive science will make for better science, fostering new discoveries and driving innovation; society as a whole will benefit.

In the course of writing this piece we were fortunate to watch ‘Picture a Scientist’, a documentary by Sharon Shattuck and Ian Cheneythe that profiles three woman scientists: Raychelle Burks, Nancy Hopkins and Jane Willenbring (see https://www.nature.com/articles/d41586-020-01912-6). The film covers some of the same ground as this essay, and is both a testament to the resilience of the three women and the stark need for the kinds of systemic changes we have argued for in this piece.

As academics and scientists we are constantly imagining the future. The policy proposals that we argue for in this article raise question: can we do this socially as well as scientifically? The fact that most institutions will now provide rooms for new mothers to breastfeed is a good story, but exemplary of institutions playing catch up with a changing society—why wasn’t this the norm twenty years ago? Rather than playing catch-up, can we proactively reimagine and then enact a modern, inclusive workplace where gender, and indeed any aspect of personal identity, is no longer a contributing factor to one’s success or failure? Flexibility, transparency, fairness and thoughtfulness will be the four core pillars of this new system.

## Supplementary information

Supplementary Information
