# Supplementary material for: Community voices: policy proposals to promote inclusion in academia through the lens of women in science
Source: Nat Commun. 2022 Jul 13;13:4068. doi: 10.1038/s41467-022-31616-6 (PMC9279379; doi:10.1038/s41467-022-31616-6)
Supplement: Supplementary file 1 — Supplementary Information [file 41467_2022_31616_MOESM1_ESM.pdf]

Supplementary Information

Personal experiences

This is a collection of our and some of our colleagues' personal experiences of being women in science.

Women in the workplace

This was said to one of us by a female colleague:

> *"You cannot be a serious scientist with those nails."*

Assumptions based on financial independence:

> *"I had an academic husband, and was asked by the department whether I needed a salary when taking a new position. The implication was that my male partner's salary would look after me, and anything else would be just 'pin money'.' This reflected an utter lack of respect for the female right to financial independence."*

Women in science repeatedly face problems relating to ambition:

> *"After seeing my male colleagues get promoted while I stayed at the same level, and knowing that our output had been totally equivalent, I asked my superior for a promotion. "I don't want you to ask for it, I want it to be a surprise," was his response. I realised I had hit this glass ceiling and so started looking elsewhere and considering offers. On telling my boss about this, he told me I was blackmailing him in order to get promoted. This was particularly remarkable and galling because I had personally witnessed male colleagues leveraging the possibility of leaving when negotiating promotions and pay rises. For them this was 'retention'; for me, 'blackmailing'."*

Being a woman in a room full of men:

> *"I remember sitting in meetings with men talking over me. Even when I said something, no one would hear me, but then when a male colleague said exactly the same thing the whole group agreed with him! It got to the point where I found a solution: whenever I wanted to get my ideas across, I went to the lab director before the meeting, had a one on one with him about the ideas, which he would then present as his own. We both knew what was happening, but in the end I didn't care about the attribution: I just wanted to get the ideas implemented."*

Being in an otherwise all-male and all-white panel where M.H. was the exception:

"After querying the make-up of the panel, I got the response from one of the panellists: 'But we've all had unconscious bias training'. It was as if they'd ticked that box and decided not to think about it again."

Conflicts of interest, independence and mentorship

An example of the negative consequences of failing to declare a romantic relationship:

"A male group leader had a romantic relationship with one of the junior women in his lab, whose PhD he was supervising. This created a clear conflict of personal interest within the line management structure of the team, and resulted in unfair treatment and credit acknowledgement for everyone in the lab. Because nothing was declared, people even started wondering if they were just imagining things, and started to doubt their own scientific competence. When news of the affair came out some years later, things seemed to make sense, but there was no consequence at all for the group leader."

Following a publication one of us received a long, personal and belittling attack email from an older male scientist (whom we had never met but knew to have a reputation for these kinds of behaviours). When such people are known to behave in such ways, the default mode in the community seems to be to hold one's hands up and say "that's just how he is" – rather than actually calling out the behaviour for what it is.

Pregnancy and childcare

One of our experiences when pregnant:

"I was a PhD student in my late twenties when I became pregnant. Early in my first trimester I informed my supervisor – "I'm sorry, I'm sure this wasn't planned," was the response. I carried on working in the lab up to eight months of pregnancy. My work involved long stretches standing on my feet, with tasks including euthanising mice with ether and dissecting embryos. Eventually, I had to be signed off work for exhaustion."

This experience is from a friend and distinguished scientist colleague of ours:

Earlier in her career she was required to attend an on-site external review of her departmental programme while on maternity leave, only a few weeks after an emergency C-section giving birth to a child with a serious condition, at a time that she was not medically permitted to drive a car. She had to give a critical presentation to an institutional quinquennial review panel. Unsurprisingly, the experience of preparing and doing the presentation and Q&A session in

front of the external panel and all her faculty peers was traumatic, and the committee's assessment was less than optimal, which was not ideal for her from a career point of view. During the whole period, she was not asked by anyone in management how she was and whether she was fit: it was simply assumed that she had to be present at this vital review.

An experience from the 2000s:

> *"I brought my baby daughter in to work with me, but we got kicked out of the office and even the shared office kitchen. The reason I was given was that having a baby in these spaces didn't comply with health and safety, but the issue was there was nowhere else to go – only the library and the canteen were deemed appropriate by the operational manager. A few years later, when the institute was designing a new building, it was encouraging to be approached by the management for feedback on how to make the new site most supportive for new mothers, which led to a dedicated private room where women can breastfeed or pump."*

For another of our colleagues, the only available option to pump was a toilet with a baby changing table.

One of us had to rely on lucky circumstances when attending a conference:

> *"I didn't want to leave my daughter when she was young, but still wanted and needed to go to conferences. Our solution was for my husband to come along with me: he could act as a nanny in the hotel room while I was in the conference. It was just a lucky coincidence, because his job meant he could work from anywhere."*
